# Zika Virus Associated Pathology and Antigen Presence in the Testicle in the Absence of Sexual Transmission During Subacute to Chronic Infection in a Mouse Model

**DOI:** 10.1038/s41598-019-44582-9

**Published:** 2019-06-06

**Authors:** Chad S. Clancy, Arnaud J. Van Wettere, John D. Morrey, Justin G. Julander

**Affiliations:** 1 Utah Veterinary Diagnostic Laboratory, School of Veterinary Medicine, Department of Animal, Dairy, and Veterinary Sciences, Utah State University, Logan, Utah 84341 United States of America; 2 Institute for Antiviral Research, Department of Animal, Dairy, and Veterinary Sciences, Utah State University, Logan, Utah 84322-5600 United States of America

**Keywords:** Epidemiology, Viral infection

## Abstract

Zika virus (ZIKV) is an arboviral infection that has been shown to be sexually transmitted. The study outlined herein aims to determine if accessory sex glands and epididymal epithelial cells are sources of viral persistence in subacute and chronic ZIKV infection, and if infection of these organs is important in sexual transmission during long-term (chronic) infection. Male interferon type I receptor knockout (*Ifnar*^*−/−*^) mice were challenged with ZIKV and reproductive tissues were harvested 14 and 35 days post infection (DPI) for inoculation studies and 14, 35 and 70 DPI for histopathology. Artificial insemination fluid derived from epididymal flush and seminal plasma from the prostate and seminal vesicle was obtained from ZIKV inoculated and sham-infected males. Naïve interferon type I and II receptor knockout (AG129) female mice were pre-treated with progesterone and inoculated intravaginally with artificial insemination fluid from ZIKV-infected males. ZIKV RNA was detected in the artificial insemination fluid generated from epididymal flush or seminal plasma from ZIKV infected males at 14 and 35 DPI. ZIKV antigens were only detected in seminiferous tubules at 14 DPI. Epididymal epithelial cells did not show ZIKV antigen immunoreactivity at 14, 35 or 70 DPI. Severe fibrosing orchitis (end stage orchitis) was observed at 35 and 70 DPI. Mild inflammation and peri-tubular fibrosis were observed in the epididymis following clearance of virus. Viral RNA was not detected by PCR in whole blood samples of females from any intravaginal experimental group and only detected in 20% of subcutaneously inoculated animals (derived from 1 experimentally infected male) at 35 DPI. While ZIKV RNA and antigens can be detected in the male reproductive tract at 14 DPI and RNA can also be detected at 35 DPI, intravaginal inoculation of artificial insemination fluid from these time-points failed to result in viremia in naïve females inoculated intravaginally. These studies support the hypothesis that epididymal epithelial cells are critical to sexual transmission in immunocompromised mice. Additionally, acute but not chronic male reproductive tract infection with ZIKV results in infectious virus capable of being sexually transmitted in mice.

## Introduction

Zika virus (ZIKV) is a unique flavivirus capable of causing both systemic and reproductive tract disease in humans. Sexual intercourse as a route of transmission within the genus *Flavivirus* has only been previously documented with Japanese Encephalitis Virus (JEV) infection in swine^1^, but the pathogenesis of reproductive tract infection and sexual transmission of disease in both ZIKV and JEV is poorly understood. Demonstration of reproductive tract infection with ZIKV has been performed independently in various animal models^2–7^. Recently, sexual transmission has been experimentally proven in a rodent model^8^ and suggested in primate models^2,7^. Epidemiologic evidence has suggested sexual transmission as an uncommon route of transmission in humans^9–13^. Though the virus is primarily transmitted through *Aedes* mosquitoes vectors^14,15^, sexual transmission of ZIKV may attribute to low-level transmission in regions that are not endemic to suitable arthropod vectors. Additionally, infection of the male reproductive tract may result in reproductive failure in humans and may cause concern to sperm bank facilities.

Epidemiologic evidence has suggested that ZIKV may be spread through sexual transmission in the ejaculate of infected men^13,16,17^. Specifically, ZIKV RNA has been demonstrated in semen of ZIKV infected men^18,19^, including vasectomized men^20^. The ejaculate of both vasectomized and non-vasectomized men was capable of causing productive infection in Vero cell culture^19^, highlighting the potential importance of the proximal vas deferens and accessory sex glands (ASG) in sexual transmission of infectious ZIKV. Interestingly, ZIKV RNA is detected in semen of men long after peripheral viremia has cleared, with persistence of RNA from 6 months to nearly 1 year reported^19,21^. There is no epidemiologic evidence to support sexual transmission during the chronic phase of reproductive tract infection in humans to date with the latest report of sexual transmission occurring at 44 days following the initiation of clinical signs^22^.

Recently, we have shown that artificial insemination fluid derived from either seminal plasma (prostatic and seminal vesicular homogenates) or epididymal flush from a ZIKV infected male mouse is capable of resulting in peripheral viremia in artificially inseminated, naïve, immunocompromised female mice^8^. These data support the hypothesis that epithelial cells of the reproductive tract support active viral replication with production of infectious virions during acute ZIKV infection. It is unknown if these epithelial cells support ZIKV infection during chronic infection or if seminiferous tubules are capable of producing infectious ZIKV during the subacute or chronic stages of disease. Additionally, it is unknown how chronic ZIKV infection impacts the physiology of the male reproductive tract in either human or animal models in terms of continued spermatic production, testosterone production and functional secretions of the ASGs.

Development of an animal model to evaluate the potential for sexual transmission from infected males is important to analyze the pathogenesis of disease and evaluate for potential therapeutic targets. Previously, we have demonstrated sexual transmission of ZIKV from seminal plasma and epididymal flush from ZIKV-infected, *Ifnar*^*−*/*−*^ (interferon type I receptor knockout) male mice to progesterone-treated, AG129 (interferon type I and II receptor knockout) female mice via artificial insemination during the acute phase of disease (7 DPI). Here, we show that ZIKV RNA persists in the artificial insemination fluid derived from epididymal flush and seminal plasma for up to 35 DPI. However, intravaginal inoculation of naïve AG129 females with artificial insemination fluid from *Ifnar*^*−*/*−*^ males at 14 and 35 DPI failed to result in systemic viremia in the naïve females. We also show that while the testicle develops a severe, irreparable, granulomatous orchitis, the epididymis and ASGs of *Ifnar*^*−*/*−*^ mice are capable of resolving ZIKV infection associated lesions in the chronic stages of disease without severe histopathologic sequelae.

## Materials and Methods

### Ethics statement

Studies were conducted under approval of the Utah State University Institutional Animal Care and Use Committee protocol number 2598. Study approval and animal care was conducted in accordance with The Guide for Care and Use of Laboratory Animals^23^ and U.S. Government Principles for the Utilization and Care of Vertebrate Animals Used in Testing, Research and Training.

### Virus

A clinical Puerto Rican isolate of ZIKV (PRVABC-59, BEI Resources, Manassas, VA, USA) was passaged in Vero 76 cells twice prior to experimental inoculation. The passaged viral stock had a titer of 10^7.0^ 50% cell culture infectious doses/mL (CCID_50_/mL).

### Mouse studies

#### Male infection and sample collection

Six to eight-week-old, male *Ifnar*^*−*/*−*^ mice were inoculated subcutaneously with either sterile minimal essential media (MEM; sham-infected; n = 19) or 10^2^ CCID_50_ of PRVABC59 ZIKV (n = 25) diluted in MEM. Samples were collected 14 (n = 9 infected; n = 8 sham-infected), 35 (n = 6 infected; n = 6 sham-infected) and 70 (n = 10 infected; n = 5 sham-infected) days post-virus infection (DPI).

The right testicle, epididymis, prostate, seminal vesicle and remaining mouse carcass were fixed in 10% neutral buffered formalin for 48 hours at room temperature. A subset of males (n = 10 infected; n = 5 sham-infected) were harvested at 70 DPI and the entire male reproductive tract was harvested for histopathologic evaluation. Tissues were incubated for 36–48 hours in 10% neutral buffered formalin.

#### Female hormonal treatment and insemination

Female 8–10-week-old, AG129 mice were pre-treated with 2 mg of progesterone (n = 50) as previously described^6,8^. Three days following progesterone administration, vaginal cytologies were prepared as previously described^8,24^.

Four insemination treatments were created (Table 1). Treatment 1 was comprised of fluid collected from epididymal flush (EF) and corresponding seminal plasma (SP) collected from a ZIKV-infected male (+; EF+, SP+ Treatment 2 was comprised of EF+ and SP from a sham-infected male (−; SP−). Treatment 3 was a positive control group composed of EF− and SP− spiked with 10^4^ CCID_50_ of cell culture derived PRVABC59 ZIKV (EF−, SP−). Treatment 4 was the negative control group was comprised of EF− and SP−.

**Table 1:** Components of each treatment group.

Treatment	Epididymal Flush (500 µL)	Seminal Plasma (250 µL)
1	Infected Male	Infected Male
2	Infected Male	Sham-infected Male
3	Sham-infected Male/Spike	Sham-infected Male/Spike
4	Sham-infected Male	Sham-infected Male

Each epididymal flush totaled 500 µl of fluid and seminal plasma totaled 250 µl of fluid.

The epididymal flush and seminal plasma fluid were combined and coincubated creating the artificial insemination inoculum.

Three replicate groups of three progesterone-treated, AG129 females were intravaginally instilled with treatments 1 and 2. One progesterone treated group of five females was assigned to treatment 3 and 4 each. Intravaginal instillation of 50 µL of insemination fluid derived from males sacrificed at 14 DPI was performed with an 18-gauge oral gavage needle. Weights and mortality of the females were recorded daily for 21 days thereafter. The treatment groups and intravaginal artificial insemination were replicated as described in the preceding paragraph for the 35 DPI male reproduction study.

#### Subcutaneous infection

As ZIKV does not routinely result in cytopathic effects in cell culture following inoculation with mouse-derived samples, a sensitive *in-vivo* model was selected to test for the presence of infectious virus in the artificial insemination fluid. The intravaginal inoculation groups were replicated as a subcutaneous inoculation in naïve AG129 mice. For the 14 DPI challenge, three females were used per challenge group. For the 35 DPI challenge, five mice (n = 3 females; n = 2 males) were used per challenge group. The challenge consisted of a subcutaneous inoculation in the inguinal area of the right hind leg of each mouse. The intravaginal inoculation fluid was diluted 1:10 in sterile MEM. A 100 µL aliquot of diluted artificial insemination solution was deposited subcutaneously in the medial aspect of the right hind limb of each mouse. Weights and mortality of the challenged mice were recorded daily for 21 days thereafter.

### Quantitative reverse transcriptase PCR

A 150 µL aliquot of insemination fluid aliquot was frozen at −80 °C for RNA isolation and quantitative reverse transcriptase PCR (qRT-PCR). At 7 DPI, 200 µL of whole blood in EDTA was collected via cheek bleed from female and male AG129 mice in both intra-vaginal and subcutaneous inoculation groups. RNA was extracted using the Qiagen QIAamp cador Pathogen Mini Kit (Qiagen, Germantown, MD, USA) according to manufacture instructions and eluted with 100 µL of elution buffer.

Quantitative RT-PCR was performed as previously described^25^ using a Mic-2 qPCR thermocycler (Bio Molecular Systems, Coomera, QLD, Australia). Standard curves of ZIKV and *GAPDH* RNA were generated with serial dilutions of synthetic RNA (GeneScript, Piscataway, NJ, USA) of the target sequence (accession HQ234499.1). The relative number of ZIKV RNA copies was determined by extrapolation from the standard curve and normalized to *GAPDH* RNA for each sample as previously described^25^.

#### Histopathology

Severity of inflammation was analyzed in the male reproductive tract of sham-infected and ZIKV-infected males at 14, 35 and 70 DPI. Male reproductive tissues were processed, embedded, and 3–5 µm thick serial sections were prepared^26^. The first slide was stained with hematoxylin and eosin and the subsequent section was used for immunohistochemistry. Two blinded veterinary anatomic pathologists (CSC and AJVW) independently scored the histologic lesion severity as previously described^25^. Statistical analysis was not performed on the male reproductive histopathologic lesion score data as currently recommended by the International Harmonization of Toxicologic Pathology Nomenclature^27^. These data are provided in full in Supplemental Tables 1 and 2.

### Immunohistochemistry

The presence of ZIKV antigen in the male reproductive tract was evaluated using immunohistochemical staining. Tissue sections were routinely deparaffinized and rehydrated. Epitope retrieval was achieved by placing slides in decloaking solution (Dako, Agilent Pathology Solutions, Santa Clara, CA), within a decloaking chamber (Biocare Medical, Pacheco, CA, USA) at 125 °C and 20 PSI for four minutes. The slides were cooled to room temperature then exposed to 0.5% triton for five minutes and washed three times in phosphate buffered saline (PBS) for 5 minutes each. Immunohistochemical reactions were performed as previously described^25^.

## Results

### Male reproductive tract histopathology and immunohistochemistry

**Figure 1:**
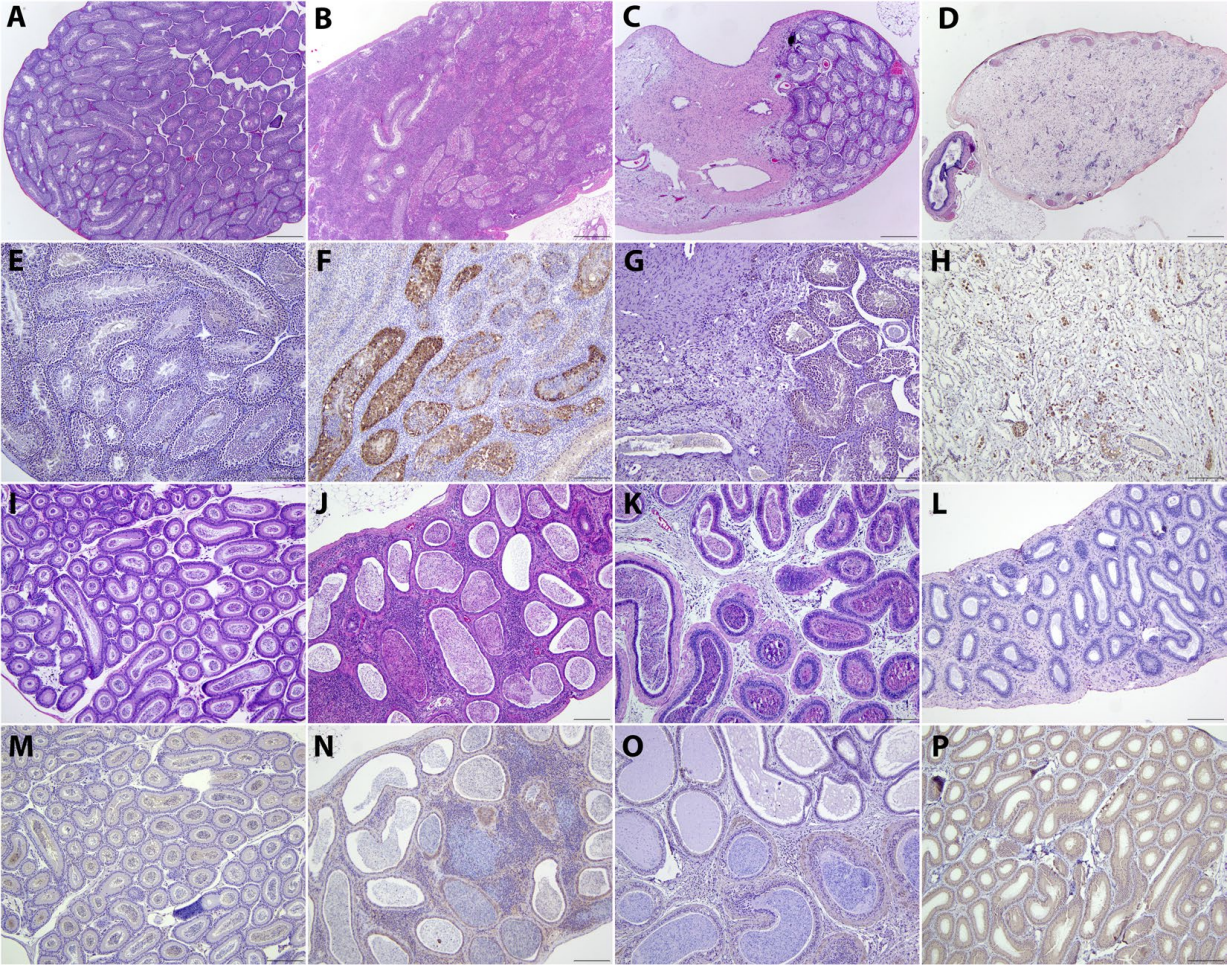
Histopathologic Progression of Zika Virus Infection in the Male Reproductive Tract. Histopathology of the testicle (**A–D**; 40×; hematoxylin and eosin stain). (**A**) Testicle of sham-infected male. Testicle of Zika Virus (ZIKV) male harvested at 14 days post infection (DPI; **B**) showing a marked inflammatory infiltrate with degeneration of seminiferous tubules that progresses to fibrosing and granulomatous orchitis by 35 DPI (**C**). Granulomatous and fibrosing orchitis is associated with seminiferous tubule degeneration that fails to resolve or regenerate by 70 DPI (end stage orchitis; **D**). Immunohistochemistry of the testicle (**E–H**; 100×; counterstained with hematoxylin). Immunohistochemical (IHC) reaction on sham-infected male testicle (**E**). ZIKV antigens are only detected in the testicle of infected males harvested 14 DPI and is associated with necrotic cellular material within the seminiferous tubules (**F**). ZIKV antigens were not detected in the testicle at 35 (**G**) or 70 DPI (**H**). Histopathology of the epididymis (**I–L**; 100×; hematoxylin and eosin stain). Epididymis of sham-infected male (**I**). The epididymis of ZIKV infected males progressed from lymphocytic infiltration with lymphoid follicle formation and epithelial cell regeneration at 14 DPI (**J**) to mild peritubular fibrosis and individual cell necrosis at 35 DPI (**K**) and moderate peritubular fibrosis with no epithelial changes at 70 DPI (**L**). IHC of the epididymis (**M**–**P**; 100×; counterstained with hematoxylin). (**M**) IHC reaction of sham-infected male epididymis. No ZIKV antigens were observed at 14 (**N**), 35 (**O**) or 70 DPI (**P**).

Epididymal inflammation was most severe at 14 DPI and was characterized by moderate to severe interstitial inflammation with rare individual epithelial cell necrosis and lymphoid nodule formation (lymphocytes, few plasma cells and histiocytes; Fig. 1N). Epididymal tubules were multifocally surrounded by a mild amount of fibrosis in all ZIKV-infected males. The lumen of epididymal tubules contained a coagulum of cellular debris and spermatids. No motile sperm were observed in the epididymal flush in the contralateral epididymis at the time of artificial insemination collection. In contrast to the epididymis, a severe neutrophilic and necrotizing orchitis was observed in the testicle at 14 DPI (Fig. 1B). ZIKV antigens were only detected in the testicle at 14 DPI, coinciding with the severe, necrotizing orchitis (Fig. 1F).

At 35 DPI, the testicle of all (n = 6) ZIKV infected males exhibited extensive fibrosis and granulomatous inflammation (Fig. 1C). Half of the infected males (n = 3) had degenerate seminiferous tubules that comprised less than 50% of the evaluated testicular area and the remaining ZIKV infected males had complete seminiferous tubule necrosis and loss. Degenerate seminiferous tubules lacked maturing spermatids. Epididymis collected at 35 DPI from ZIKV infected males consistently exhibited multifocal interstitial fibrosis and rare individual epithelial cell necrosis (Fig. 1G). Multifocal tubules exhibited epithelial regeneration characterized by increased cellular cytoplasmic basophilia and stratification of the plump epithelium. Sham-infected (n = 6) males did not exhibit epididymal or testicular pathology at any time point. ZIKV antigens were not detected in the epididymis at 14 or 35 DPI nor in the testicle at 35 DPI (Fig. 1N,O,G respectively). ZIKV antigens were not detected in any sham-infected male at any time point (Fig. 1E,M).

To assess the potential for seminiferous tubule recovery, a subset of male mice were sacrificed at 70 DPI, at the presumed completion time of two full spermatic cycles. A severe fibrosing orchitis (end stage orchitis) with histiocytic and lymphocytic inflammation was observed in all ZIKV-infected males (n = 10) at this time point (Fig. 1D). No seminiferous tubules were observed in any male at this time point. Additionally, interstitial fibrosis and intra-luminal cellular debris without discernable spermatids was observed in the epididymis of all ZIKV infected males at 70 DPI. Immunohistochemistry did not reveal ZIKV antigens in the testicle or epididymis at 70 DPI (Fig. 1H).

**Figure 2:**
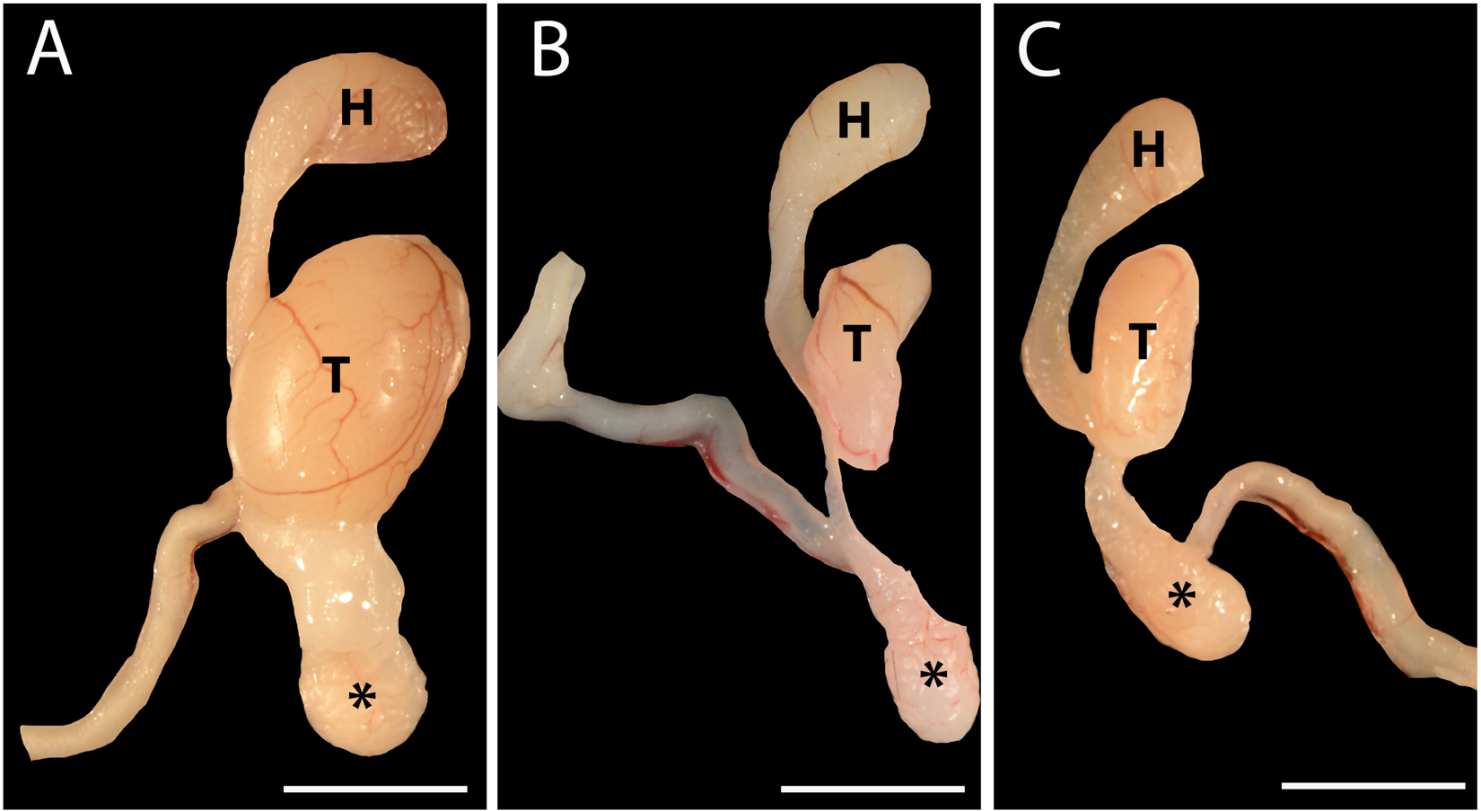
Gross Pathology of Testicles at 35 and 70 Days Post Infection (DPI). (**A**) Testicle, epididymis and vas deferens of sham-infected male. (**B**) Testicle, epididymis and vas deferens from Zika virus (ZIKV) infected male at 35 DPI. There is a significant decrease in the testicular mass and the vas deferens is translucent. (**C**) Testicle, epididymis and vas deferens from ZIKV male at 70 DPI showing similar gross pathology as males at 35 DPI. H = head of epididymis; T = testicle; *tail of epididymis; Size bar = 5 mm.

On gross examination of the male reproductive tract (Fig. 2), there was an obvious decrease in testicular size at 35 (2B) and 70 DPI (2C) relative to sham-infected males. Additionally, the vas deferens at 35 and 70 DPI was translucent. Histologically, the translucent vas deferens corresponded with absence of mature spermatids.

Mild prostatic inflammation characterized by predominantly mononuclear cell infiltration was observed in the stroma abutting the prostate in 33% (n = 3/9) of ZIKV infected males and 11% (n = 1/9) of sham-infected males at 14 DPI. Similarly, peri-prostatitis was observed in 50% (n = 3/6) of ZIKV infected males and 20% (n = 1/5) of sham-infected males at 35 DPI. Inflammation in the seminal vesicle and the bulbourethral gland were not observed in either ZIKV infected or sham-infected group at either 14 or 35 DPI.

### Artificial insemination fluid quantitative RT-PCR results

**Figure 3:**
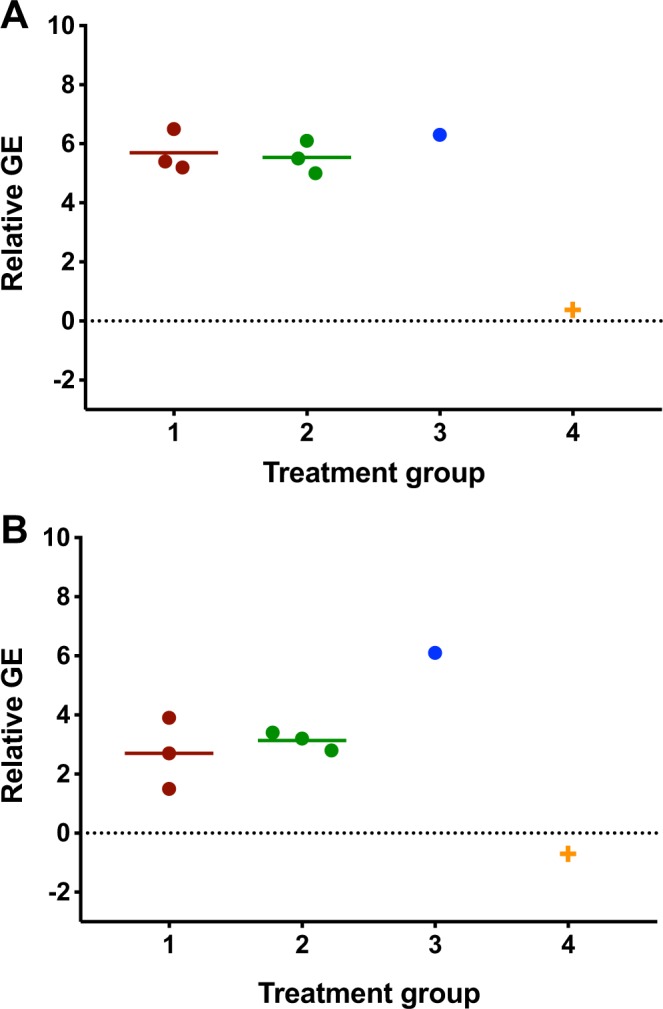
Relative genome equivalents of Zika virus (ZIKV) RNA in Artificial Insemination Fluid. Viral RNA was detected in both experimental inoculation group artificial insemination fluid media at both 14 (**A**) and 35 (**B**) DPI with a relatively higher viral titer observed at 14 DPI. Treatment 1: ZIKV infected epididymal flush and seminal plasm, treatment 2: ZIKV infected epididymal flush, sham-infected seminal plasma, treatment 3: sham-infected epididymal flush and seminal plasma spike with ZIKV; treatment 4: sham-infected epididymal flush and seminal plasma: GE = genome equivalents.

ZIKV RNA was detected in the artificial insemination fluid derived from male mice experimentally inoculated with ZIKV on both 14 and 35 DPI (Fig. 3). ZIKV RNA was not detected in the sham-infected, non-spiked artificial insemination fluid from either 14 or 35 DPI. A substantial drop in relative genome equivalents of viral RNA between 14 and 35 DPI was observed in experimentally infected males (Fig. 3).

### Experimental inoculation disease parameters

**Figure 4:**
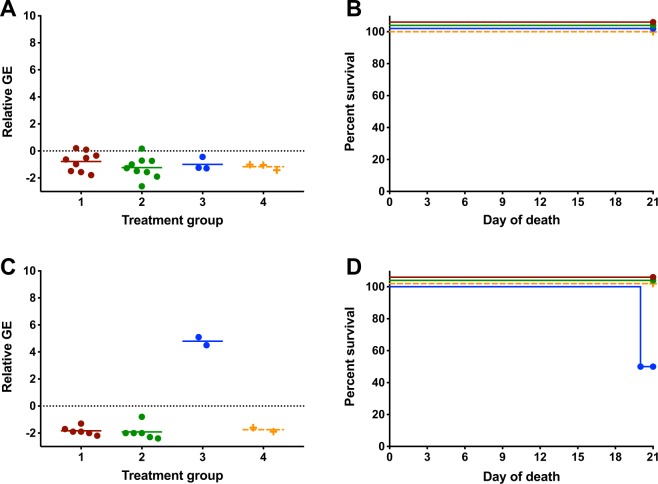
Experimental Inoculation Disease Parameters for 14 days Post Inoculation Cohort. Viremia was not detected in either experimental inoculation treatment in either intravaginal or subcutaneously inoculated females at 14 DPI (**A**,**C**). Viremia and mortality were observed in subcutaneously inoculated females from treatment 3, from which artificial insemination fluid was spiked with cultured ZIKV. Treatment 1: ZIKV infected epididymal flush and seminal plasm, treatment 2: ZIKV infected epididymal flush, sham-infected seminal plasma, treatment 3: sham-infected epididymal flush and seminal plasma spike with ZIKV; treatment 4: sham-infected epididymal flush and seminal plasma: GE = genome equivalents.

Morbidity, mortality and peripheral viremia were evaluated for both intravaginal and subcutaneous experimentally inoculated mice. ZIKV RNA was not detected in peripheral blood samples in any intravaginally inoculated females that were artificially inseminated using samples collected from ZIKV infected males at the 14 DPI time point (Fig. 4A). Similarly, 100% survival was observed to 21 DPI in all intravaginal inoculation groups from 14 DPI males (Fig. 4B). Corresponding subcutaneous inoculation showed peripheral viremia in 100% (n = 2) of the positive control (treatment 3) group (Fig. 4C). ZIKV RNA was not detected in any other females challenged subcutaneously with day 14 samples. A singular female in treatment 3 (50%, n = 1) from the subcutaneous inoculation met early euthanasia criteria at 20 DPI and both females in treatment 3 (EP−/SF− spiked with ZIKV) exhibited severe weight loss over the course of the disease (Fig. 4D).

**Figure 5:**
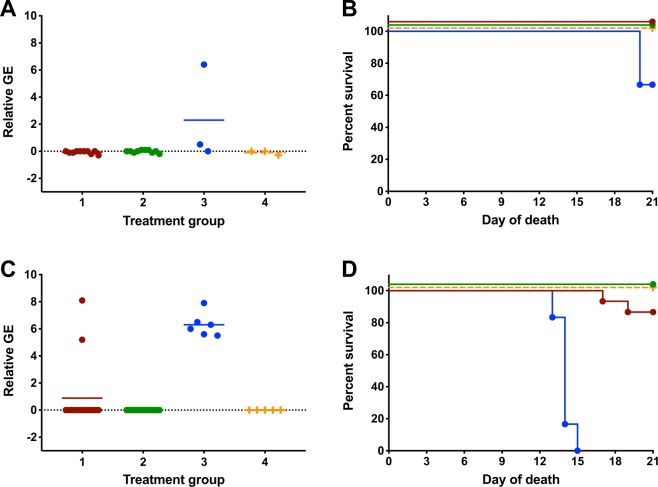
Experimental Disease Parameters for 35 Days Post Inoculation Cohort. Peripheral viremia was observed in a subset of animals from treatment three, artificial insemination fluid from sham-infected males spiked with Zika virus, in both intravaginal and subcutaneous infections (**A**,**C**). Rare (n = 2) animals had peripheral viremia from treatment one (EF+/SP+) when the artificial insemination fluid was deposited subcutaneously (**C**). EF = epididymal flush; SP = seminal plasma Treatment 1: ZIKV infected epididymal flush and seminal plasm, treatment 2: ZIKV infected epididymal flush, sham-infected seminal plasma, treatment 3: sham-infected epididymal flush and seminal plasma spike with ZIKV; treatment 4: sham-infected epididymal flush and seminal plasma: GE = genome equivalents.

Intravaginal inoculation of naïve females using 35 DPI male time point insemination fluid resulted in no detectable ZIKV RNA in peripheral blood samples in treatments 1, 2 or 4 (Fig. 5A). In the ZIKV spiked treatment (treatment 3), 33% of females (n = 1/3) developed a viral load of approximately 6.0 relative genome equivalents and required euthanasia at 20 DPI. All other females in the intravaginal inoculation group survived to 21 DPI (Fig. 5B). In corresponding subcutaneous studies, 100% of mice (n = 5) in treatment 3 (EP−/SF− spiked with ZIKV) had detectable peripheral viremia (Fig. 5C) that progressed rapidly to meet early euthanasia criteria by 13–15 DPI (Fig. 5D). Two mice in treatment 1 (EF+/SP+; n2/5; 13%) had detectable peripheral ZIKV RNA at 7 DPI and met early euthanasia criteria at 17–19 DPI. No viremia, morbidity or mortality was observed in subcutaneously inoculated mice in treatments 2 (EF+/SP−) and 4 (EF−/SP−).

## Discussion

Zika virus is an emerging arbovirus in the Western hemisphere that has been shown to be sexually transmitted. For sexual transmission to occur, a primary infection of the male or female reproductive tract must occur with subsequent release of infectious virions in reproductive secretions. Previously, we have shown that epididymal epithelial cells are a likely source of infectious virions during the acute phase of male reproductive tract disease, and ASGs may also serve as a source of infectious virus^8^. Here we show that antigens are detectable in the testes during subacute (14 DPI) but not chronic (35 and 70 DPI) infection. ZIKV antigens are not detected in epididymal cells during subacute or chronic infection. These results suggest that viral antigen is either absent or expressed at levels below the limit of detection during chronic infection. Additionally, ZIKV antigens were not detected in the ASGs (prostate and seminal vesicle) during the course of disease in this study. We also show that while ZIKV RNA is present in the artificial insemination fluid during subacute and chronic infection, this RNA largely represents non-infectious virus. Infectious virus was detected on 35 DPI in artificial insemination from one infected male when the fluid was inoculated subcutaneously. These data suggest that sexual transmission could potentially occur and that infectious virus, although rare, may be observed in chronically infected *Ifnar*^*−*/*−*^male mice. While most sexual transmission occurs within the first weeks of initial exposure in humans, chronic infection may be capable of resulting in low level transmission^10,11,17,22,28,29^.

We corroborate the previously published data that ZIKV can infect the seminiferous tubules of the testicle resulting in a robust inflammatory response leading to an end-stage, fibrosing and granulomatous orchitis^5,30–32^. Orchitis was first observed at 14 DPI and was associated with ZIKV antigens in both Sertoli cells and degenerate cells within the seminiferous tubules. As previous studies have shown, testicular inflammation in the mouse model is non-reparable^32^. Here, we show that the reduction in testicular size is due to granulomatous inflammation and fibrosis rather than simple atrophy as previously reported^32^. We show that at two complete spermatic cycles following peripheral infection, there is no evidence of seminiferous tubule regeneration, even in the absence of ZIKV antigen. While immunocompromised mice progress from a severe neutrophilic and necrotizing orchitis to an end-stage, granulomatous and fibrosing orchitis, this condition is unlikely to occur in natural infection in human males. There is no current clinical data that suggests orchitis on physical examination of infected men, and there is a lack of scrotal or testicular pain associated with presenting complaints of ZIKV positive men.

In contrast to the testicle, the epididymis rapidly clears ZIKV antigens and has limited chronic inflammation in this immunocompromised mouse model. Previously, inflammation and antigen presence has been characterized in the epididymis during the acute phase of disease^25^. The epididymis has been hypothesized to serve as the source of infectious virus not only during sexual transmission to the naïve recipient, but also to naïve tissue in the male reproductive tract, chiefly the testicle^8^. In this study, ZIKV antigens were not observed during either subacute (14 DPI) or chronic (35 and 70 DPI) infection. Importantly, sexual transmission was not observed within the study, suggesting that active infection of epididymal epithelial cells plays a crucial role in sexual transmission of ZIKV. Limited data currently exists in animal models evaluating the antigen presence and progression of pathology associated with ZIKV infection in the epididymis^31^. Here, we show that while pathology progresses in the epididymis with inter-tubular/peri-tubular fibrosis and lymphoid follicle formation within the stroma, the epididymal epithelium is capable of regeneration following ZIKV infection and lacks expression of ZIKV antigens during the chronic phase of reproductive tract infection. The inflammatory response observed throughout ZIKV infection in immunocompromised male mice may recapitulate the reproductive tract disease that occurs in humans. However, epididymitis is also not reported clinically with ZIKV infection, which suggests that the extent of pathology that occurs in male mouse models may be more severe than what occurs in humans. While the histopathologic lesions observed in the male mouse epididymis may be more severe than what occurs in human disease, male mice are capable of resolving acute ZIKV disease in the epididymal epithelium within two full spermatic cycles. These data suggest that male reproductive tract lesions in humans may be temporary and reparable, resulting in full physiologic function following acute disease.

Minimal and inconsistent pathology was observed in the ASGs on histologic examination. Additionally, ZIKV antigens were not detectable at any time point evaluated during the study. It is important to note that lymphocytic prostatitis was noted in 1 sham-infected control male at each time point and mild lymphocytic prostatitis was considered an insignificant, background lesion in this study population. It is unknown if the mild increase in the prevalence in prostatitis observed in the ZIKV infected males is due to ZIKV infection as limited sample size does not provide enough statistical power for evaluation. There was no difference in ability for ZIKV to be transmitted via artificial insemination between groups in which seminal plasma was harvested from ZIKV infected males versus seminal plasma from sham-infected males. These data suggest that ZIKV does not cause significant subacute or chronic disease in the ASG. Previous studies have shown immunomodulatory effects of seminal plasma within the female reproductive tract with both stimulatory and suppressive effects published^33–36^. Previously, we hypothesized that ZIKV infection of ASG may alter the secretions of ASG epithelial cells during acute and chronic infection, which may enhance sexual transmission of disease. ZIKV does not appear to alter ASG physiology to create a more susceptible environment for sexual transmission during the subacute and chronic phase of male reproductive tract disease. The epithelial cells of the ASGs are not a likely source of viral persistence in the male reproductive tract as neither antigen nor infectious virus was routinely observed in seminal plasma of experimentally infected males.

Even with detectable viral RNA in the artificial insemination fluid, female mice failed to develop viremia following intra-vaginal inoculation with artificial insemination fluid derived from males at 14 or 35 DPI. This supports clinical data from ZIKV infected men in which virus could be cultured from the ejaculate on cells during the acute phase of disease (days to weeks), but not during the chronic phase of disease (weeks to months), though viral RNA persisted^19^. Fourteen DPI coincides with the first detectable presence of ZIKV antigen within the seminiferious tubules in our *Ifnar*^*−*/*−*^ mouse model. Interestingly, epididymal epithelial cells have no detectable ZIKV antigens at this time point. These data highlight that infection of epididymal epithelial cells, and not the seminiferous tubules, are important in the pathogenesis and sexual transmission of ZIKV. It is unknown how RNA is maintained long-term in the reproductive tract without detection of infectious virions. Potential hypotheses could include production and secretion of a neutralizing IgA antibody within the epididymal or vas-deferent mucosa, detection of RNA within apoptotic or necrotic epithelial cells within the reproductive tract, or faulty production of virions following acute infection of the epithelial cells. Lack of antigen detection in epididymal epithelial cells from 14–70 DPI would suggest either shedding of RNA in necrotic/apoptotic cellular debris or faulty viral production following acute phase of disease as the most likely hypotheses. Deep-sequencing of the artificial insemination fluid to detect potential variations in the viral RNA or identify potential defective interfering particles may aid in further evaluation of this anomaly.

We have previously hypothesized and shown that epithelial cells from either the epididymis or the ASGs (prostate and seminal vesicle) serve as potential sources of infectious virus in the male reproductive tract during acute infection. Here, we show that epithelial cells within the epididymis lose immunoreactivity by 14 DPI and remain free of ZIKV antigens up to two spermatic cycles (70 DPI). However, seminiferous tubules within the testicle are highly immunoreactive to ZIKV antigens at 14 DPI and rapidly lose immunoreactivity by 35 DPI. In our mouse model, there is no correlation between ZIKV antigens in the testicle or testicular pathology, and sexual transmission of disease. Taken in context with previously published data^8^, infection of the epididymal epithelial cells during acute infection is likely responsible for production of infections virions. Infection of the testicle does not appear to play a primary role in the sexual transmission of ZIKV in this immunocompromised mouse model. This is a critical model difference as ZIKV results in a severe fibrosing (end-stage) orchitis in susceptible, immunocompromised mouse strains. Together, these studies show that future work evaluating for antiviral therapeutics and sexual transmission is best performed on immunocompromised mouse strains (i.e. AG129 and *Ifnar*^*−*/*−*^) males during the acute phase of disease around 7 DPI. Additionally, these data suggest that future work to evaluate the pathogenesis of virus from viremia to infection of epididymal epithelial cells is necessary.

## Supplementary information

Supplemental Table 1
