# Supplementary material for: Zika Virus Associated Pathology and Antigen Presence in the Testicle in the Absence of Sexual Transmission During Subacute to Chronic Infection in a Mouse Model
Source: Sci Rep. 2019 Jun 6;9:8325. doi: 10.1038/s41598-019-44582-9 (PMC6554467; doi:10.1038/s41598-019-44582-9)
Supplement: Supplementary file 1 — Supplemental Table 1 [file 41598_2019_44582_MOESM1_ESM.pdf]

Zika Virus Associated Pathology and Antigen Presence in the Testicle in the Absence of Sexual Transmission During Subacute to Chronic Infection in a Mouse Model

Chad S. Clancy[1,2], Arnaud J. Van Wettere[1], John D. Morrey[2], Justin G. Julander[2]

[1]Utah Veterinary Diagnostic Laboratory, School of Veterinary Medicine, Department of Animal, Dairy, and Veterinary Sciences, Utah State University, Logan, Utah, 84341, United States of America

[2]Institute for Antiviral Research, Department of Animal, Dairy, and Veterinary Sciences, Utah State University, Logan, Utah, 84322-5600, United States of America

Supplemental Table 1

Days Post Infection	Testicle (%; x/n)				Epididymis (%; x/n)			
	None	Mild	Moderate	Severe	None	Mild	Moderate	Severe
14	0 (0/9)	0 (0/9)	11 (1/9)	89 (8/9)	0 (0/9)	22 (2/9)	67 (6/9)	11 (1/9)
35	0 (0/6)	0 (0/6)	0 (0/6)	100 (6/6)	NA	NA	NA	NA

Histopathologic Score of Testicular and Epididymal Inflammation. NA= not applicable

Supplemental Table 2

Peri-tubular Fibrosis (%; x/n)	Epithelial Necrosis (%; x/n)	Interstitial Epididymitis (%; x/n)
100 (6/6)	83 (5/6)	33 (2/6)

Characteristics of Epididymal Inflammation at 35 Days Post Infection.
